# Managing Recurrent Clozapine-Induced Constipation in a Patient with Resistant Schizophrenia

**DOI:** 10.1155/2021/9649334

**Published:** 2021-11-08

**Authors:** Silviu Tomulescu, Kim Uittenhove, Reda Boukakiou

**Affiliations:** ^1^Hôpitaux Universitaires de Genève, Rue Gabrielle-Perret-Gentil 4, 1205 Genève, Switzerland; ^2^Université de Genève, 24, Rue du Général-Dufour CH, 1211 Genève, Switzerland

## Abstract

Clozapine is an effective antipsychotic for the treatment of resistant schizophrenia. However, clozapine can lead to serious side effects. One of the most common side effects is constipation and in rare cases ileus, which is associated with a considerable case fatality rate. Our patient exhibited repeated episodes of ileus while being treated with clozapine. We adapted the treatment of the patient in several ways to manage these severe side effects. First, we reduced clozapine dosage by opting for an augmentation strategy of clozapine through paliperidone. Then, we added linaclotide as a nonconventional laxative. We further adapted treatment after the occurrence of a volvulus prompting surgical intervention which revealed a malformation of the intestines' peritoneal attachment. A gastrostomy to facilitate the treatment of any further episode was performed and bethanechol was introduced alongside linaclotide. Follow-up revealed the efficacy of our strategy involving the use of linaclotide in managing the side effects of clozapine in this patient.

## 1. Introduction

Clozapine is the first synthetised atypical antipsychotic [1] and is recommended for the treatment of resistant schizophrenia [2] but is linked to serious adverse effects like agranulocytosis, seizures, cardiovascular effects, or gastrointestinal effects [3]. Clozapine is the antipsychotic with the most potent anticholinergic effect [4], an effect that is dose-dependent [5], and precipitates constipation in 14% to 60% of patients [6, 7]. In some cases, treatment with clozapine has been linked to ileus. A Chinese population study on 7921 patients treated with clozapine found that 8 cases of ileus were reported [8]. Ileus is a serious side effect and leads to many fatalities, with Palmer establishing a 27% case fatality rate in a group of 102 patients who were exhibiting gastrointestinal side effects under clozapine treatment [9]. The onset of ileus occurs on average four years after starting clozapine, indicating that the side effect is more associated with the maintenance phase than the introductory period [10]. Due to the high fatality rate related to these side effects, clozapine is reserved for adults suffering from resistant schizophrenia as discussed by Kane et al., for whom no treatment alternatives can be found. Schizophrenia is classified as resistant when the patient has not adequately responded to two trials of different neuroleptics [1].

Here, we present a case report on the management of severe and resistant ileus in a patient treated with clozapine for resistant schizophrenia with different strategies, including the use of linaclotide. To our knowledge and at the time of writing of this paper, there are no case studies in the scientific literature on the use of linaclotide to counter clozapine-induced constipation [11].

## 2. Case Report

Mr. T. is a middle-aged, unmarried man who lives in a nursing home. He has been dependent all his life because of moderate intellectual disability due to neonatal hypoxemia. He was diagnosed with schizophrenia during his teenage years with delusions, auditory verbal hallucinations, disorganized behavior, and avolition. He has been hospitalised over 30 times in inpatient settings due to multiple psychotic episodes with psychomotor agitation and aggression. The patient has undergone multiple trials of neuroleptics, from the moment of his diagnosis onwards.

In March 2008, he developed a neuroleptic malignant syndrome following the association of amisulpride and levomepromazine, which prompted a brief hospitalisation in the intensive care unit. Afterwards, the medical team opted for treatment with clozapine, given that the patient resisted the previous trials with different antipsychotics. In December 2008, he was discharged and was prescribed 425 mg/day of clozapine, which shows effectiveness in preventing relapse.

In July 2015, the patient was hospitalised for ileus for the first time. He underwent an exploratory laparoscopy, which was converted to a laparotomy, but no lesion could be found. Clozapine-induced paralytic ileus 3was diagnosed. Given the fatality risk associated with the side effects in this patient, clozapine was replaced by paliperidone 9 mg/day. Following a psychotic relapse, the dose was increased in April 2016 to 12 mg/day. However, another psychotic episode occurred one month later, in May 2016, and prompted the reinstauration of 400 mg/day clozapine. Since then, no psychotic relapse occurred. However, the patient suffered further episodes of constipation, such as a 4-day streak without stool in September 2016. In January 2017, while receiving 450 mg/day of clozapine, the patient developed a new episode of ileus. A new laparotomy was performed, which only cleared the adhesions. Because of the danger of psychotic relapse in this patient, clozapine treatment was maintained and led to 2 more episodes of ileus (in March and May 2017), where lubiprostone was unsuccessfully administrated and which were resolved by conservative treatment.

The clozapine posology was left unchanged and the patient was admitted to our Complex Intervention Unit (Unité de Psychiatrie Hospitalière Adulte, UPHA) in 2018, with a new episode of paralytic ileus. The surgery team opted for a conservative treatment—gastric evacuation by nasogastric intubation and fasting. Meanwhile, we continued oral drug administration. Our strategy for managing clozapine-induced ileus in this patient consisted of several steps. First, given that research shows that these side effects are dose-dependent [3], we aimed to reduce the dosage of clozapine to the minimum while preserving efficacy. The impaired absorption by the aspirative nasogastric intubation coupled with clinical stability from a psychiatric point of view allowed us to determine the minimally effective plasmatic concentration of clozapine. In partnership with the patient and his family representative, we furthermore initiated a clozapine potentiation strategy by adding paliperidone with daily body temperature monitoring and weekly blood test including creatinine kinase levels, to prevent a neuroleptic malignant syndrome. This step allowed us to diminish the dose of clozapine while maintaining efficacy: the patient was stable under 175 mg of clozapine and 6 mg of paliperidone. The treatment was well tolerated. We attempted to further reduce the dosage to 150 mg of clozapine and 6 mg of paliperidone, but at this dosage, the patient showed early cues of a psychotic episode that were clinically assessed (insomnia, irritability, and behavioural symptoms such as yelling). It is important to note that, during the time of the psychotropic medication adjustment, the patient did not have spontaneous stool and received an enema every 2 days.

Our next step involved addressing the constipation issue directly. Given the history of lack of efficacy of laxative treatment with lubiprostone, we opted for a novel treatment with linaclotide 290 *μ*g preparation. Note that although this is recognized as a potential treatment in managing clozapine-induced constipation, no data are currently available on its use [11]. Treatment with linaclotide enabled the patient to have spontaneous but watery, diarrheic stools, which occurred during the nights. We switched the administration schedule of the drug from morning to bedtime and reduced the dosage to 72 *μ*g. With this adapted schedule and dosage, 1-2 more concentrated stools would occur during daytime, allowing the patient to return to his institution.

Following discharge, no further ileus episode occurred for 18 weeks. After this period, the patient developed a volvulus. The surgery team reported that the mechanism responsible for the volvulus was a congenital default where the small bowel is not attached to the posterior abdominal cavity wall, causing it to tangle under its own weight, citing their intraoperatory finding of atone, dilated bowel. To prevent further volvulus episodes, they created a gastrostomy. On the medical side, we further introduced bethanechol, a cholinergic agent that we used off-label to reduce constipation and titrated up to a dose of 200 mg a day. Following administration of bethanechol, we measured the abdominal perimeter which decreased from a baseline of 106 cm to 86 cm (19% reduction), testifying to the efficacy of bethanechol in addition to linaclotide.

Thus, the patient returned to his residency with a treatment of clozapine 175 mg/day, paliperidone 6 mg/day, bethanechol 200 mg/day, and linaclotide 72 *μ*g/day and having spontaneous stools. Furthermore, we put into place a protocol for the caretakers, consisting of enemas, to be administered if no stools would occur for 24 hours and/or the abdominal perimeter is larger than 98 cm.

The one-year follow-up revealed that the patient developed two more episodes of volvulus, rapidly resolved by the surgery team, who used the gastrostomy tube to pump air. No more psychotic episodes nor ileus occurred.

## 3. Discussion

**Figure 1:**
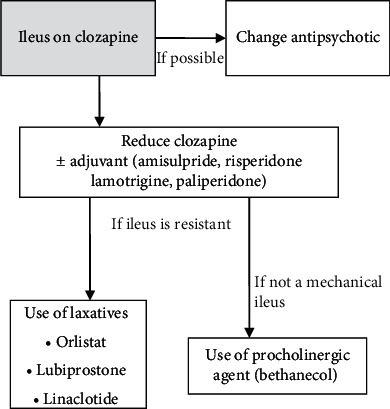
Treatment algorithm for the management of an ileus on clozapine.

In this patient, we successfully managed clozapine-induced constipation by applying a combination of steps (Figure 1). In the first step, we attempted to reduce constipation issues by decreasing the dosage of clozapine (175 mg/day), while maintaining treatment efficacy through a paliperidone (6 mg/day) augmentation strategy. In the second step, we added laxatives specifically targeted at relieving constipation. We opted for a novel but promising laxative, consisting of linaclotide (72 *μ*g/day). Finally, our patient presented additional complexities, which we addressed with a final prescription of a procholinergic agent, bethanechol (200 mg/day).

We will now review each of these steps and strategies as a function of the literature and our experience related to this case report.

### 3.1. Reducing Clozapine Dosage or Changing to a Different Antipsychotic

Many antipsychotics do not have the serious side effects that clozapine does. However, our patient had a history of treatment-resistant schizophrenia, and attempts to change to a different antipsychotic were followed by psychotic relapse. Therefore, our first step was to maintain clozapine treatment and to decrease the dose in order to alleviate anticholinergic side effects. This step involved an augmentation. The most effective adjuvants to clozapine mentioned in the literature are amisulpride [12], lamotrigine [13], and paliperidone [14]. We excluded the use of amisulpride, as the patient had a precedent of neuroleptic malignant syndrome in response to this antipsychotic. Between lamotrigine and paliperidone, we opted for the latter because of several reasons: (1) a relatively fast crosstitration period compared to lamotrigine, (2) the patient had previously received paliperidone without reported adverse effect, and (3) paliperidone has no antagonist activity at cholinergic receptors and thus theoretically a low propensity to cause anticholinergic adverse effects.

The association between clozapine and paliperidone enabled us to decrease clozapine dosage by more than 50% without psychotic relapse. However, constipation persisted, which prompted the next step in our treatment.

### 3.2. Use of Laxatives

Our next step was to add laxatives. The literature describes the use of two laxatives for treating clozapine-induced constipation: orlistat and lubiprostone.

Orlistat is a weight-control molecule that inhibits lipase and is the only nonconventional laxative studied with a randomized placebo-controlled trial for treatment of clozapine-induced constipation. The results showed that clozapine-induced constipation was reduced from 50% at baseline to 20% at week 4 [15]. In our case, we did not use orlistat given the patient was underweight (objectified by the BMI at 17 and the low prealbumin level), having lost more than 10 kg in a short period following conservative treatment of ileus which implied fasting and nasogastric aspiration.

Lubiprostone is a fatty acid derivative of the E1 prostaglandin which increases the intestinal secretion. This drug is marketed for idiopathic constipation and constipation associated with IBS. With regard to the treatment of clozapine-induced constipation, Meyer and Cummings [16] mention the use of lubiprostone at 24 mcg/day along with docusate and lactulose in a case report.

In our case, we had to exclude both types of laxative. We did not prescribe orlistat because our patient was underweight (BMI at 17 and low prealbumin level) and had lost more than 10 kg in a short period of time following conservative treatment of ileus through fasting and nasogastric aspiration. We also did not use lubiprostone, since this medication had previously proven ineffective in our patient in a three-week trial during a previous ileus episode.

Therefore, we opted for a new class of laxatives, including linaclotide, a peptide which augments the intestinal secretion through the activation of guanylate cyclase C. This medication is currently used for chronic idiopathic constipation [17]. To this day, no data or case reports document the use of linaclotide in managing clozapine-induced constipation [11]. Upon introducing linaclotide and adapting the administration schedule and dosage, our patient produced frequent and solid stools. Over two weeks, the stools hardened from 7 to 5 on the Bristol stool scale as per the clinical experience with the product [18].

### 3.3. Use of a Procholinergic Agent

Bethanechol is a cholinergic agonist breveted in 1943 and used for urinary retention. It was first mentioned by Everett [19] in 1975, who prescribed doses of 25 mg 3 times a day to treat anticholinergic side effects of tricyclic antidepressants. Poetter and Stewart published a case report [20] on the use of bethanechol 30 mg/day for clozapine-induced constipation. They reported that the results were satisfactory, although the patient continued to take senna syrup daily and received two lidocaine/docusate enemas a week.

Initially, we did not prescribe bethanechol due to the presence of a contraindication: mechanical ileus in the medical history of our patient. However, in our patient, intraoperatory findings and the absence of adherences confirmed paralytic ileus and not mechanical ileus. Therefore, we were able to additionally prescribe this molecule, with good results, evident in the reduction of the abdominal perimeter. In the first hospitalisation, we did not use bethanechol due to its absolute contraindication: mechanical ileus in the medical history.

## 4. Conclusion

Managing clozapine-induced constipation is a complicated matter. Different strategies must be considered and potentially combined to minimize risks and maximize benefits. The ideal treatment plan will crucially depend on the medical history and risk factors associated with each patient.

Lastly, our case report shows that linaclotide is a powerful laxative which should be considered part of a treatment plan for clozapine-induced constipation. A careful harm-benefit weighing is imperative, and all strategies must be considered and, if possible, discussed with the patient.

A recent paper published shows a significant improvement in patients with clozapine-induced constipation with prucalopride, a 5-HT4 receptor agonist [21], showing interest in the use of new laxatives.
